# *Ganoderma tsuage* promotes pain sensitivity in aging mice

**DOI:** 10.1038/s41598-024-61499-0

**Published:** 2024-05-21

**Authors:** Kai-Ning Yang, Chia-Ying Lin, Wei-Nong Li, Chao-Ming Tang, Jyotirmayee Pradhan, Ming-Wei Chao, Chia-Yi Tseng

**Affiliations:** 1 Taoyuan General Hospital, Ministry of Health and Welfare, Taoyuan, Taiwan; 2 Department of Biomedical Engineering, Chung Yuan Christian University, Taoyuan, Taiwan; 3 Department of Bioscience Technology, Chung Yuan Christian University, Taoyuan, Taiwan

**Keywords:** Ganoderma, Antioxidant, Hypoalgesia, Aging, Neuroscience, Neurology

## Abstract

Advances in modern medicine have extended human life expectancy, leading to a world with a gradually aging society. Aging refers to a natural decline in the physiological functions of a species over time, such as reduced pain sensitivity and reaction speed. Healthy-level physiological pain serves as a warning signal to the body, helping to avoid noxious stimuli. Physiological pain sensitivity gradually decreases in the elderly, increasing the risk of injury. Therefore, geriatric health care receives growing attention, potentially improving the health status and life quality of the elderly, further reducing medical burden. Health food is a geriatric healthcare choice for the elderly with *Ganoderma tsuage* (GT), a Reishi type, as the main product in the market. *GT* contains polysaccharides, triterpenoids, adenosine, immunoregulatory proteins, and other components, including anticancer, blood sugar regulating, antioxidation, antibacterial, antivirus, and liver and stomach damage protective agents. However, its pain perception-related effects remain elusive. This study thus aimed at addressing whether *GT* could prevent pain sensitivity reduction in the elderly. We used a galactose-induced animal model for aging to evaluate whether *GT* could maintain pain sensitivity in aging mice undergoing formalin pain test, hot water test, and tail flexes. Our results demonstrated that *GT* significantly improved the sensitivity and reaction speed to pain in the hot water, hot plate, and formalin tests compared with the control. Therefore, our animal study positions *GT* as a promising compound for pain sensitivity maintenance during aging.

## Introduction

According to the definition of the World Health Organization (WHO), a society is considered "aging”, “aged”, and “super-aged” when the proportion of its elderly population aged over 65 years reaches 7%, 14%, and 20% of the total population, respectively^1^. WHO statistical data points out that the ratio of the elderly population over the age of 65 years is expected to grow from 10% in 2022 to 16% in 2050^2^. Therefore, the long-term care for the elderly is becoming increasingly important. Aging is accompanied by several chronic diseases, the most common being pain sensitivity deterioration^3^.

Multiple studies demonstrated that pain sensitivity reduction is a common problem in the elderly. In the experiment of David and Eugene in 1960^4^, 90 young men and women (aged 20–30 years) and 110 elderly people (aged 65–97 years) of the same community were stimulating their forehead by a heat source using a projector bulb to form a light through a lens. Gradually increasing the stimulation number, frequency, and intensity, the researchers observed eye muscle contractions and recorded the pain description of the subjects. The study demonstrated reduced pain perception sensitivity and relative response in the elderly^4^. Further studies also linked aging and pain sensitivity^5–8^. There are also experiments comparing old and young mice, revealing that old mice also exhibit reduced pain sensitivity^9–11^.

Reshi, a mushroom type of the *Ganoderma* genus, has been used as medicine for thousands of years in China, Korea, Japan, and other Asian regions. Its metabolites, polysaccharides, and terpenoids are functional ingredients for multiple disease prevention and treatment^12^ with anticancer, blood sugar regulatory, antioxidation, antibacterial, antiviral, and liver and stomach protective properties^13–19^. Among the different Reshi types, only red, purple, and spruce ones are officially recognized for their medical effects with *Ganoderma tsugae* (GT) being the best antioxidant^20,21^.

Although pain is a subjective feeling that cannot be directly expressed, it could still be judged from human and animal behavioral responses, including escape, dodging, and other reactions to eliminate painful stimuli^22^. The pain test in mice is mainly used to evaluate analgesic effects. A standard pain test approach involves pain recognition by observing the licking response in mice to a hot plate^23–25^, a tail-flick reaction by hot water^24,26^ or thermal radiation^25,27^, and formalin assay^24,25^. Accordingly, this study aimed at investigating whether GT would exhibit a protective effect on aging-induced pain sensitivity reduction. To the best of our knowledge, no previous study has demonstrated the correlation between GT and pain perception so far, underlining the importance of our study.

## Materials and methods

### Animals and treatments

We used 5-week-old male ICR mice (BioLASCO Taiwan) in this study, approved by the Institutional Animal Care and Use Committee of Chung Yuan Christian University (Approved protocol no# 105016). The animals were kept under the following conditions: 18–26 °C, 30–70% humidity, and a 12:12 h light–dark cycle with free access to laboratory chow and water throughout the study. We divided the animals into four groups as follows: Control (Control), GT treatment (GT), d-galactose (d-gal) treatment (aging), and d-gal plus GT treatment (aging + GT). In the d-gal treatment group, we subcutaneously administered daily d-gal injections (100 mg/kg)^28^ to the mice for eight weeks. In the d-gal plus GT treatment group, GT (200 μg/kg)^28^ was administered as oral gavage daily concomitantly with D-gal injections for eight weeks. All control animals were subcutaneously administered phosphate-buffered saline at equal volumes. In the GT treatment group, oral gavage was performed daily for eight weeks. At the end of the final week, we performed the behavior tests with the mice, then sacrificed them and conducted biochemical analyses. The behavior tests are described in detail below. The mice were sacrificed with carbon dioxide inhalation in the original feeding box, which was placed in the euthanasia container. Carbon dioxide was gradually filled the euthanasia container at a rate of 20–30% of the chamber volume per minute, causing the animal to lose consciousness quickly. Carbon dioxide was continuously perfused for another five minutes after the animal appeared dead. If mice showed anxiety during the sacrifice, 4–5% isoflurane was given to induce an anesthetic effect that caused them to lose consciousness before the carbon dioxide perfusion. Mice’s death was confirmed before removing it from the euthanasia container. All methods were carried out in accordance with relevant guidelines and regulations of NIH Guide for the Care and Use of Laboratory Animals, and all methods are reported in accordance with Animal Research: Reporting of In Vivo Experiments (ARRIVE) guidelines^29^.

### Ganoderma tsugae extraction

GT samples were gifts from Professor Ruey-Shyang Hseu, Department of Biochemical Science and Technology, National Taiwan University. Dr. Hseu entrusted the Li-Kang Biotechnical Co., Ltd (I-Lan, Taiwan) to culture and collect the mycelium. He further extracted the GT from the fruiting body using hot water as described previously^30^. Briefly, the fruiting body was homogenized in sterilized water. The collected sample was frozen at − 20 °C, vacuum-dried for 36 h, then stored at − 20 °C until further use. Dr. Hseu provided and authorized us to use the water extract for this study. The crude mixture contained 1.96% triterpenes and 3.93% polysaccharides^31^. The dried extract was further analyzed for the total water-soluble polysaccharide, total triterpenoid, beta-d-glucan, and heavy metal contents. For total water-soluble polysaccharides, the sample was extracted, the polysaccharides were precipitated with ethanol, re-dissolved, reacted with phenol–sulfuric acid, and analyzed under a UV–Vis Spectrophotometer (UV–Vis) (ChromTech® CT-2200, Taiwan). The amount of total water-soluble polysaccharides was 45.4 g/100 g. For total triterpenoids, the sample was extracted, reacted with a vanillin-glacial acetic acid-perchloric acid solution as a color-developing agent, and analyzed under a UV–Vis. We registered the amount of total triterpenoids as 463 mg/100 g. Concerning beta-d-glucans, the sample was dissolved to remove low-molecular-weight sugar, hydrolyzed by enzyme specificity, and analyzed using a High-Performance Anion Exchange Chromatography-Pulsed Amperometric Detector (HPAEC-PAD) (Thermo Fischer Scientific, Germering, Germany). The amount of beta-D-glucans was 0.24 g/100 g. Finally, we detected no heavy metals. The GT water extraction was followed by DMSO precipitation, reserve dialysis, and protein depletion. The final triterpenoid and water-soluble polysaccharide content was 128 and 0.39 g/100 g in GT-DMSO solution, respectively. Our previous preliminary study followed a GT dosage of 200 μg/kg/day^32^.

### Open-field test (OPT)

Open-field test was performed based on the previous publication with modification^33^. An acrylic box measuring approximately 100 × 100 × 30 cm was divided by gridlines into nine equally sized squares that served as nine zones. Each mouse was placed individually in the central square. Crossing was defined as exiting from the previous square with all four paws. Between the tests, the acrylic box was wiped with 75% alcohol to avoid odor interference. Crossing frequency and duration spent in the center square within five minutes was recorded for each mouse. Behavioral data were acquired by video recording and software analysis (Noldus Information Tech, Ethovision XT 10.0, Wageningen).

### Tail-flick test-hot water

The tail-flick test-hot water, in general pain-related experiments, is mostly a one-time test for analgesic evaluations^24,26^. Briefly, mice were put in the restrainer, exposed, and soaked the rear 2/3 tail in a constant temperature water bath at 55 °C. The time was recorded from tail soaking to flicking. In order to avoid scalding the tail, the soaking time did not exceed 15 s. If no tail flicking occurred in 15 s, we stopped scalding and removed the mouse.

### Tail-flick test-hot plate

The hot plate test is performed based on the previous report with modifications^34^. The thermal stimulation was performed using the hot plate (Panlab model LE7406, Harvard Apparatus, Holliston, MA). First, we set the hot plate tester to 55 °C, and when the temperature reached 55 °C, we put the mouse into the acrylic cover to measure the time it licked its feet. If no reaction was given in 90 s, we removed the mouse from the hot plate to prevent tissue damage and recorded the time as 90 s.

### Formalin pain test

The formalin pain test follows the process^35,36^: The mice were placed in a transparent 9.5 cm-diameter and 10 cm-tall jar for 5 min to adapt to the environment, followed by a 20 μL 2% formalin injection into the right hind paw. We recorded the number of mice licking their feet between 0 and 10 min after the injection (pain stimulation) and the time between 10 and 30 min (inflammatory response of the surrounding tissue).

### Statistical analysis

The data are represented as the mean ± standard error. The statistical significance (*p* < 0.05) was determined using the GraphPad InStat software 3.05 package for Windows (GraphPad Software Inc., San Diego, CA).

## Results

### No significant change in the physiological value of the mice in different groups

**Figure 1:**
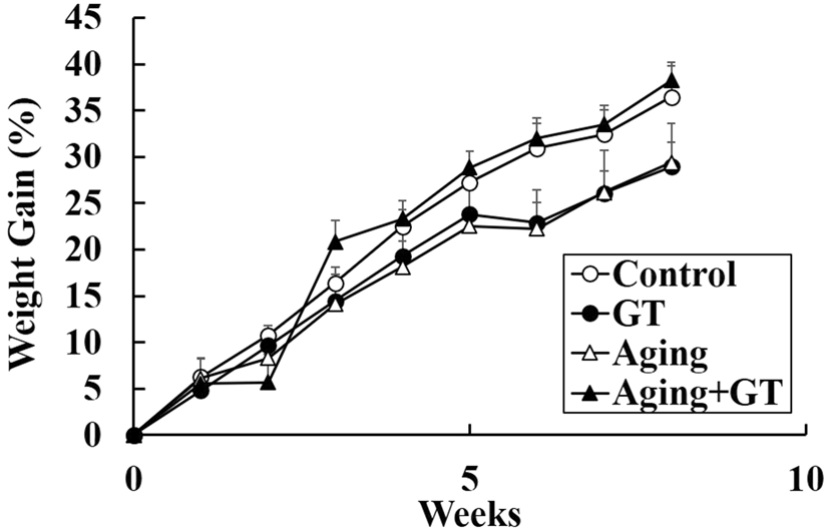
Body weight changes in mice during the experiment. The body weight increased healthily in each group with no significant differences. N = 6 except control (N = 5).

First, we measured and recorded the body weight and basic hematology values of the mice, detecting a healthy weight increase each week with no significant difference between the GT-treated and control animals (Fig. 1). Moreover, the hematology results in mice demonstrated that the white blood cell (WBC), red blood cell, and hemoglobin values fell in the healthy range in all groups with no significant differences, although aging mice displayed higher WBC numbers (Table 1).

**Table 1:** Changes in physiological parameters of ICR.

Groups	Parameters*			
	Control	GTDE	D-gal	D-gal + GTDE
Hemoglobin (g/dL)	12.76 ± 0.59	11.30 ± 0.50	12.97 ± 1.07	13.67 ± 0.93
RBC (10^9^/mL)	5.38 ± 0.42	5.69 ± 0.95	6.24 ± 1.64	6.37 ± 2.03
WBC (10^6^/mL)	4.01 ± 0.27	4.15 ± 0.41	5.72 ± 0.91	4.49 ± 0.68

*The differences in hemoglobin level, RBC count, and WBC count were not statistically significant. The physiological parameters were compared using the one-way ANOVA followed by the Tukey–Kramer multiple comparisons test. Means ± SEM, n = 6.

### D-gal and GT have no significant effect in open-field test

**Figure 2:**
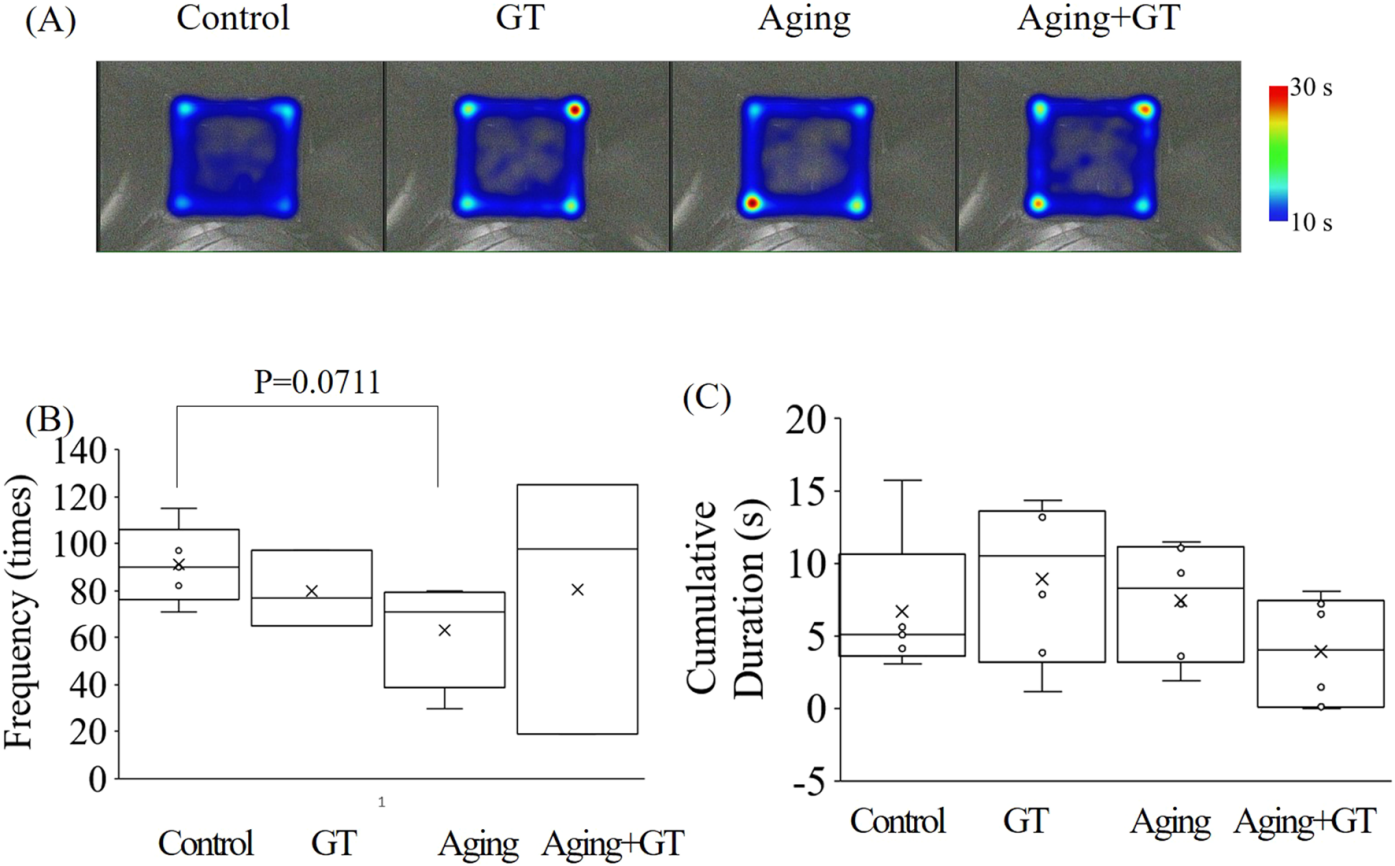
Open-field test, indicating no difference in locomotion between the groups. (**A**) Heat map diagrams between the groups. Warm and cold colors refer to longer and shorter stay in the area, respectively. (**B**) Frequency quantification of passing the center in each group. The data indicates a lower frequency in the aging group compared to the control with a nearly significant difference. (**C**) Cumulative duration of staying in the center area with no differences among the groups. N = 6 except control (N = 5).

The OPT evaluates alertness in mice. In general, mice are more alert to new environments, they thus tend to move along corners and walls and are less likely to walk into open fields (e.g., the middle of a box). Our data revealed that mice in each group stayed less in the central quadrant (Fig. 2A). The mobility of mice could be judged by their movement frequency in each quadrant and accumulative time in the field (Fig. 2B,C). Compared with the control, the movement frequency of the aging group displayed a downward trend, near the level of significance (Fig. 2B). In contrast, the GT group was more active than the aging group, although no significant difference could be observed among the groups. Finally, no significant difference could be detected between the groups for a cumulative duration either (Fig. 2C).

### GT gavage improves the reaction time in the Tail-flick test against hot water stimulation

**Figure 3:**
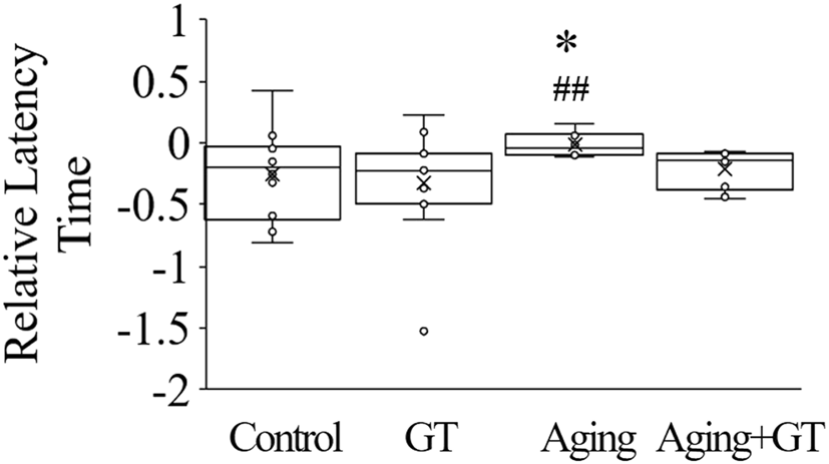
GT gavage prevents slow reaction times in D-gal-induced aging mice during the tail-flick pain sensitivity test upon hot water stimulation. The results confirmed that the GT-fed and aging groups displayed a faster and slower reaction speed, respectively. *p < 0.05 compared to the control and ^##^*p* < 0.01 compared to the GT group by Student’s t-test. n = 2 (2 tests/mice), N = 6 except control (N = 5).

We applied the hot water pain test to assess the response time of mice to painful stimulations. We subtracted the data of week 0 from that of each week to reveal a potential increase or decrease in the reaction times relative to week 0 before D-gal induction and GT gavage. Negative and positive values indicated shorter and longer reaction times (i.e., faster and slower reactions) in a given week compared to week 0, respectively. Overall, the reaction time in the control group slightly reduced and that in the GT group reduced even more, indicating shorter reaction times required for the tail-flick reaction in mice compared to week 0 (Fig. 3). Moreover, we observed no significant difference between these two groups. However, the reaction time in the aging group significantly increased, which could be significantly reduced by GT, bringing it closer to the standard reaction time.

### GT prevents the D-gal-induced increase in reaction time in the tail-flick test by hot plate stimulation test

**Figure 4:**
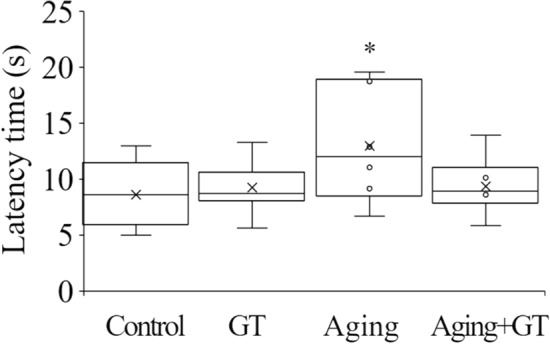
D-gal-induced aging reduced traction in the tail-flick test for pain sensitivity upon hot plate stimulation. Compared with the control, reaction time of the aging group significantly increased, and it recovered upon GT administration. *p < 0.05 compared to the control using Student’s t-test. N = 6 except control (N = 5).

The paw-licking response in mice was tested using a hot plate apparatus. Our results demonstrated that the reaction time of the aging group was significantly longer compared to the control (Fig. 4). Upon GT administration, the reaction time returned to the level of that in the control, displaying no significant difference.

### GT prevents the D-gal-induced pain reaction in the formalin test

**Figure 5:**
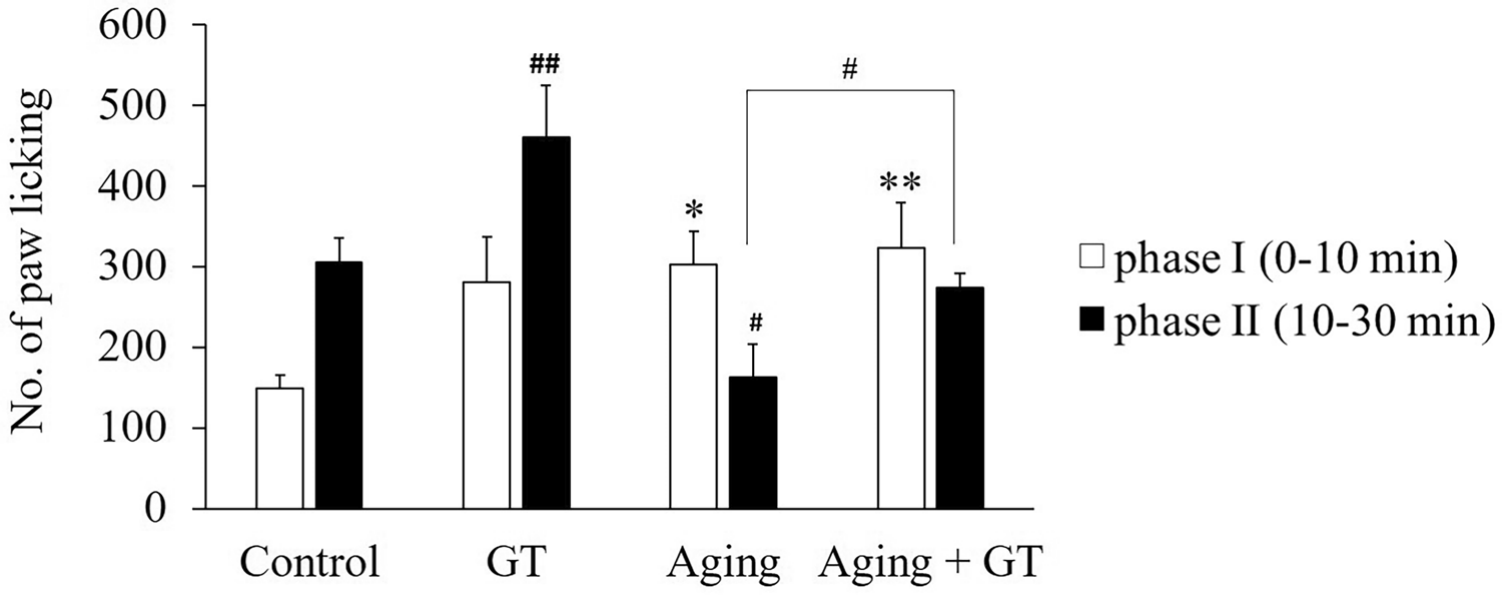
Formalin test to assess pain reaction in the right paw of mice upon D-gal-induced aging. In phase I (0–10min), the number of mice licking the paw increased in the D-Gal aging group compared to the control. In phase II (10–30min), the number of paw licking in the GT group significantly increased compared with the control. The number of paw-licking events in the aging group significantly decreased, which could be reversed by GT. *p < 0.05, **p < 0.01 compared to the control of phase I using Student’s t-test and ^#^*p* < 0.05, ^##^*p* < 0.01 compared to the control of phase II by Student’s t-test. N = 6 except control (N = 5).

The foot licking response in mice upon formalin injection could be divided into two intervals for analysis and judgment. In phases I (0–10 min) and II (10–30 min), the number of paw-licking events in the D-Gal treatment group increased and decreased compared with the control, respectively (Fig. 5). Moreover, both in phase I and phase II, the number of paw lickings significantly increased in the GT group. Besides, in phase I, GT treatment has no significant effect on the paw licking between the aging + GT group and the aging group. However, in phase II, paw-licking events significantly increased and recovered to the control level.

## Discussion

The weight of the mice increased healthily during the experiment without any excessive stress or sudden weight loss due to the experimental operation, drug administration, and induced aging (Fig. 1). The field results revealed two main behaviors: alertness and activity. Since mice exhibit better alertness to their environment in general, they would avoid exploring unknown areas (the central quadrant) and move mostly along the walls^37,38^. Induced aging and GT administration did not change the alertness in mice (Fig. 2A,C). However, moving frequency between each quadrant, which could be considered as an locomotor activity indicator in mice, decreased in mice of the aging group (Fig. 2A,C). Previous studies underlined that aging might lead to reduced locomotor activity in mice^7,39^, which is in good agreement with our experimental results and hints that our induction of aging was successful. Among the experimental groups that with induced aging and GT administration exhibited better mobility than the induced aging-only group. Therefore, we could imply that galactose administration could indeed trigger aging in mice and reduce their mobility^40,41^, and GT could prevent this phenomenon.

Concerning the reaction time in hot water, while the aging group required longer reaction time than the control^42^, GT administration improved pain sensitivity in mice, thereby reducing the reaction time to the control level (Fig. 3). Furthermore, the hot plate-related results for paw-licking reaction testing demonstrated that aging mice needed a longer reaction time following hot plate stimulation, thereby confirming that pain sensitivity is lower in aging mice^43,44^. This phenomenon could be prevented by GT administration, indicating that GT could contribute to maintaining a healthy pain sensitivity system.

The response to the formalin test could be divided into two main periods and analyzed separately. Higher numbers of paw-licking events indicate higher-level pain sensation in mice. In phase I (0–10 min), the pain response is caused by a foreign matter invading the sole, the number of paw lickings thus increased in the GT, aging, and aging + GT treatment groups. In phase II (10–30 min), the pain is caused by inflammation^35,45–47^, leading to significantly higher numbers in paw-licking events in the GT treatment group compared to the control (Fig. 5). The d-gal aging groups were significantly lower than the control group, but the number of paw lickings in the aging + GT group has recovered. Based on phase II, GT administration could increase pain sensitivity in mice. Aging reportedly reduces pain sensitivity^48^. Human studies also showed that pain sensitivity is decreased with aging^3,8^, while other groups show opposite results^46^. Moreover, data have shown that elderly subjects appear to be more susceptible to long-term pain, and drugs that target peripheral sensitization are less effective^46^. Therefore, according to our animal study, GT could be a potent treatment to overcome aging-induced pain sensitivity reduction.

The limitation of our study is that we only report GT’s effect on males. Literature, both in humans and rodents, shows that females have a lower pain threshold. Therefore, the pain sensitivity in females is higher than in males^49–53^. One researcher has the opposite opinion: From the point of view of the dentist, women have a higher tolerance to pain than men^54^. However, the literature illustrated that pain sensitivity depends on the type of pain source: Some show female sensitivity higher than males^49–53^, while others show no differences^55,56^. Even more, some reports show that female has a different tolerance to pain in the hormone cycle^57–59^. Moreover, for the same reason, males show higher reactivity and effect than females to the pain-killing drug or related chemicals treatment^53,60^. Thus, males have a more stable reaction to the pain sensation and therapy. We chose male mice for our model first. Accordingly, in the future direction of our study, we will not only look at the GT effect against pain in both genders, but we might also examine it in different degrees of aging.

## Conclusion

This is the first study applying Reishi in reduction of pain sensitivity during aging. The results of this animal study demonstrated that aging reduces pain sensitivity, which could be potentially restored by GT in mice, thereby providing a potential candidate to solve the problem of reduced pain sensitivity against aging.

## Supplementary Information

Supplementary Information.

## Data Availability

Data is provided within the manuscript or Supplementary Information files.

## References

[1] Lin, Y. Y. & Huang, C. S. Aging in Taiwan: Building a society for active aging and aging in place. *Gerontologist* **56**(2), 176–183 (2016). 26589450 10.1093/geront/gnv107

[2] Gerland, P. What's beneath the future: World population prospects. In *Semaine Data-SHS* (2023).

[3] Lautenbacher, S. *et al.* Age changes in pain perception: A systematic-review and meta-analysis of age effects on pain and tolerance thresholds. *Neurosci. Biobehav. Rev.* **75**, 104–113 (2017). 28159611 10.1016/j.neubiorev.2017.01.039

[4] Sherman, E. D. & Robillard, E. Sensitivity to pain in relationship to age. *J. Am. Geriatr. Soc.* **12**, 1037–1044 (1964). 14217863 10.1111/j.1532-5415.1964.tb00652.x

[5] Covinsky, K. E. *et al.* Pain, functional limitations, and aging. *J. Am. Geriatr. Soc.* **57**(9), 1556–1561 (2009). 19682122 10.1111/j.1532-5415.2009.02388.x PMC2925684

[6] Gagliese, L. Pain and aging: The emergence of a new subfield of pain research. *J. Pain* **10**(4), 343–353 (2009). 19327641 10.1016/j.jpain.2008.10.013

[7] Tinnirello, A., Mazzoleni, S. & Santi, C. Chronic pain in the elderly: Mechanisms and distinctive features. *Biomolecules* **11**, 8 (2021). 10.3390/biom11081256 PMC8391112 34439922

[8] Riley, J. L. *et al.* Age and race effects on pain sensitivity and modulation among middle-aged and older adults. *J. Pain* **15**(3), 272–282 (2014). 24239561 10.1016/j.jpain.2013.10.015 PMC4005289

[9] Omar, N. M. *et al.* Influence of age on pain sensitivity in response to paw pressure and formalin injection in rats: A role of nitric oxide. *Gen. Physiol. Biophys.* **31**(2), 185–194 (2012). 22781822 10.4149/gpb_2012_023

[10] Legg, E. D., Novejarque, A. & Rice, A. S. The three ages of rat: The influence of rodent age on affective and cognitive outcome measures in peripheral neuropathic pain. *Pain* **144**(1–2), 12–13 (2009). 19464801 10.1016/j.pain.2009.04.023

[11] Sadler, K. E. *et al.* Central amygdala activation of extracellular signal-regulated kinase 1 and age-dependent changes in inflammatory pain sensitivity in mice. *Neurobiol. Aging* **56**, 100–107 (2017). 28526294 10.1016/j.neurobiolaging.2017.04.010 PMC5497765

[12] Paterson, R. R. M. Ganoderma—A therapeutic fungal biofactory. *Phytochemistry* **67**(18), 1985–2001 (2006). 16905165 10.1016/j.phytochem.2006.07.004

[13] Wang, M. & Yu, F. Research progress on the anticancer activities and mechanisms of polysaccharides from ganoderma. *Front. Pharmacol.* **13**, 891171 (2022). 35865946 10.3389/fphar.2022.891171 PMC9294232

[14] Liu, Q. & Tie, L. Preventive and therapeutic effect of ganoderma (Lingzhi) on diabetes. *Adv. Exp. Med. Biol.* **1182**, 201–215 (2019). 31777020 10.1007/978-981-32-9421-9_8

[15] Wang, C. *et al.* Triterpenes and aromatic meroterpenoids with antioxidant activity and neuroprotective effects from *Ganoderma lucidum*. *Molecules* **24**, 23 (2019). 10.3390/molecules24234353 PMC6930543 31795252

[16] Seweryn, E., Ziala, A. & Gamian, A. Health-promoting of polysaccharides extracted from *Ganoderma lucidum*. *Nutrients* **13**, 8 (2021). 10.3390/nu13082725 PMC8400705 34444885

[17] Cor Andrejc, D., Knez, Z. & Knez Marevci, M. Antioxidant, antibacterial, antitumor, antifungal, antiviral, anti-inflammatory, and nevro-protective activity of *Ganoderma lucidum*: An overview. *Front. Pharmacol.* **13**, 934982 (2022). 35935849 10.3389/fphar.2022.934982 PMC9353308

[18] Park, J. H. *et al.**Ganoderma lucidum* pharmacopuncture for the treatment of acute gastric ulcers in rats. *J. Pharmacopunct.* **17**(3), 40–49 (2014). 10.3831/KPI.2014.17.025 PMC4332011 25780708

[19] Qiu, Z., Zhong, D. & Yang, B. Preventive and therapeutic effect of ganoderma (Lingzhi) on liver injury. *Adv. Exp. Med. Biol.* **1182**, 217–242 (2019). 31777021 10.1007/978-981-32-9421-9_9

[20] Yen, G.-C. & Wu, J.-Y. Antioxidant and radical scavenging properties of extracts from *Ganoderma tsugae*. *Food Chem.* **65**(3), 375–379 (1999). 10.1016/S0308-8146(98)00239-8

[21] Mau, J. L., Lin, H. C. & Chen, C. C. Antioxidant properties of several medicinal mushrooms. *J. Agric. Food Chem.* **50**(21), 6072–6077 (2002). 12358482 10.1021/jf0201273

[22] Mauderli, A. P., Acosta-Rua, A. & Vierck, C. J. An operant assay of thermal pain in conscious, unrestrained rats. *J. Neurosci. Methods* **97**(1), 19–29 (2000). 10771071 10.1016/S0165-0270(00)00160-6

[23] Porreca, F. *et al.* Roles of mu, delta and kappa opioid receptors in spinal and supraspinal mediation of gastrointestinal transit effects and hot-plate analgesia in the mouse. *J. Pharmacol. Exp. Therap.* **230**(2), 341–348 (1984). 6086883

[24] Couto, V. M. *et al.* Antinociceptive effect of extract of *Emilia sonchifolia* in mice. *J. Ethnopharmacol.* **134**(2), 348–353 (2011). 21185930 10.1016/j.jep.2010.12.028

[25] Chu, C. *et al.* Anti-nociceptive activity of aqueous fraction from the MeOH extracts of *Paederia scandens* in mice. *J. Ethnopharmacol.* **118**(1), 177–180 (2008). 18456440 10.1016/j.jep.2008.03.011

[26] Cui, J. *et al.* Effect of needling “Housanli” (ST 36) with different retaining-needle time on the pain threshold of mice using the hot water tail-flick test. *Chin. Acupunct. Moxibust.* **29**(8), 653–654 (2009). 19947272

[27] D’amour, F. E. & Smith, D. L. A method for determining loss of pain sensation. *J. Pharmacol. Exp. Therap.* **72**(1), 74–79 (1941).

[28] Kuo, H. C. *et al.**Ganoderma tsugae* prevents cognitive impairment and attenuates oxidative damage in d-galactose-induced aging in the rat brain. *PLoS ONE* **17**(4), e0266331 (2022). 35390035 10.1371/journal.pone.0266331 PMC8989198

[29] Percie du Sert, N. *et al.* Reporting animal research: Explanation and elaboration for the ARRIVE guidelines 2.0. *PLoS Biol.* **18**(7), e3000411 (2020). 32663221 10.1371/journal.pbio.3000411 PMC7360025

[30] Liang, Z.-C., Hseu, R.-S. & Wang, H.-H. Partial purification and characterization of a 1,3-β-d-glucanase from *Ganoderma tsugae*. *J. Ind. Microbiol.* **14**(1), 5–9 (1995). 10.1007/BF01570058

[31] Tseng, C. Y. *et al.* Potent in vitro protection against PM(2.5)-caused ROS generation and vascular permeability by long-term pretreatment with *Ganoderma tsugae*. *Am. J. Chin. Med.* **44**(2), 355–76 (2016). 27080945 10.1142/S0192415X16500208

[32] *Abstracts from the 4th Global Chinese Symposium and the 8th Symposium for Cross-Straits, Hong Kong and Macao on Free Radical Biology and Medicine.* Chin. Med., 2018. **13**(2): p. 56.

[33] Gould, T. D., Dao, D. T. & Kovacsics, C. E. The open field test. In *Mood and Anxiety Related Phenotypes in Mice: Characterization Using Behavioral Tests* (ed. Gould, T. D.) 1–20 (Humana Press, 2009).

[34] Bannon, A. W. & Malmberg, A. B. Models of nociception: Hot-plate, tail-flick, and formalin tests in rodents. *Curr. Protoc. Neurosci.* **8**, 9 (2007). 10.1002/0471142301.ns0809s41 18428666

[35] Liu, X. *et al.* Formalin-induced and neuropathic pain altered time estimation in a temporal bisection task in rats. *Sci. Rep.* **9**(1), 18683 (2019). 31822729 10.1038/s41598-019-55168-w PMC6904569

[36] Tjolsen, A. *et al.* The formalin test: An evaluation of the method. *Pain* **51**(1), 5–17 (1992). 1454405 10.1016/0304-3959(92)90003-T

[37] Rosso, M. *et al.* Reliability of common mouse behavioural tests of anxiety: A systematic review and meta-analysis on the effects of anxiolytics. *Neurosci. Biobehav. Rev.* **143**, 104928 (2022). 36341943 10.1016/j.neubiorev.2022.104928

[38] Sturman, O., Germain, P. L. & Bohacek, J. Exploratory rearing: A context- and stress-sensitive behavior recorded in the open-field test. *Stress* **21**(5), 443–452 (2018). 29451062 10.1080/10253890.2018.1438405

[39] Sprott, R. L. & Eleftheriou, B. E. Open-field behavior in aging inbred mice. *Gerontology* **20**(3), 155–162 (1974). 10.1159/000212009 4461327

[40] Firdaus, Z. *et al.**Centella asiatica* prevents D-galactose-Induced cognitive deficits, oxidative stress and neurodegeneration in the adult rat brain. *Drug Chem. Toxicol.* **45**(3), 1417–1426 (2022). 33078641 10.1080/01480545.2020.1833907

[41] Belviranli, M. & Okudan, N. Coconut oil ameliorates behavioral and biochemical alterations induced by D-GAL/AlCl(3) in rats. *Brain Res.* **1823**, 148704 (2024). 38052316 10.1016/j.brainres.2023.148704

[42] Mitzelfelt, J. D., Carter, C. S. & Morgan, D. Thermal sensitivity across ages and during chronic fentanyl administration in rats. *Psychopharmacology (Berl.)* **231**(1), 75–84 (2014). 23900640 10.1007/s00213-013-3208-4 PMC3858394

[43] Raut, A. & Ratka, A. Oxidative damage and sensitivity to nociceptive stimulus and opioids in aging rats. *Neurobiol. Aging* **30**(6), 910–919 (2009). 17997197 10.1016/j.neurobiolaging.2007.09.010 PMC2742467

[44] Shoji, H. & Miyakawa, T. Age-related behavioral changes from young to old age in male mice of a C57BL/6J strain maintained under a genetic stability program. *Neuropsychopharmacol. Rep.* **39**(2), 100–118 (2019). 30816023 10.1002/npr2.12052 PMC7292274

[45] Dubuisson, D. & Dennis, S. G. The formalin test: A quantitative study of the analgesic effects of morphine, meperidine, and brain stem stimulation in rats and cats. *Pain* **4**, 161–174 (1977). 564014 10.1016/0304-3959(77)90130-0

[46] Pepino, L. *et al.* Formalin-evoked pain triggers sex-specific behavior and spinal immune response. *Sci. Rep.* **13**(1), 9515 (2023). 37308519 10.1038/s41598-023-36245-7 PMC10261048

[47] Wang, L. *et al.* The antinociceptive properties of the *Corydalis yanhusuo* extract. *PLoS ONE* **11**(9), e0162875 (2016). 27622550 10.1371/journal.pone.0162875 PMC5021270

[48] Scuteri, D. *et al.* Effects of aging on formalin-induced pain behavior and analgesic activity of gabapentin in C57BL/6 mice. *Front. Pharmacol.* **11**, 663 (2020). 32457634 10.3389/fphar.2020.00663 PMC7227482

[49] Canete, T. & Gimenez-Llort, L. Preserved thermal pain in 3xTg-AD mice with increased sensory-discriminative pain sensitivity in females but affective-emotional dimension in males as early sex-specific AD-phenotype biomarkers. *Front. Aging Neurosci.* **13**, 683412 (2021). 34354580 10.3389/fnagi.2021.683412 PMC8329418

[50] Fillingim, R. B. *et al.* Sex, gender, and pain: A review of recent clinical and experimental findings. *J. Pain* **10**(5), 447–485 (2009). 19411059 10.1016/j.jpain.2008.12.001 PMC2677686

[51] Skovbjerg, S. *et al.* Conditioned pain modulation and pressure pain sensitivity in the adult Danish general population: The DanFunD study. *J. Pain* **18**(3), 274–284 (2017). 27884690 10.1016/j.jpain.2016.10.022

[52] Doehring, A. *et al.* Effect sizes in experimental pain produced by gender, genetic variants and sensitization procedures. *PLoS ONE* **6**(3), e17724 (2011). 21423693 10.1371/journal.pone.0017724 PMC3053372

[53] Pieretti, S. *et al.* Gender differences in pain and its relief. *Ann. Ist Super Sanita* **52**(2), 184–189 (2016). 27364392 10.4415/ANN_16_02_09

[54] Wesolowicz, D. M. *et al.* The roles of gender and profession on gender role expectations of pain in health care professionals. *J. Pain Res.* **11**, 1121–1128 (2018). 29942147 10.2147/JPR.S162123 PMC6007196

[55] Racine, M. *et al.* A systematic literature review of 10 years of research on sex/gender and experimental pain perception—Part 1: Are there really differences between women and men? *Pain* **153**(3), 602–618 (2012). 22192712 10.1016/j.pain.2011.11.025

[56] Edwards, R. R. *et al.* Effects of gender and acute dental pain on thermal pain responses. *Clin. J. Pain* **15**(3), 233–237 (1999). 10524477 10.1097/00002508-199909000-00011

[57] Hellstrom, B. & Lundberg, U. Pain perception to the cold pressor test during the menstrual cycle in relation to estrogen levels and a comparison with men. *Integr. Physiol. Behav. Sci.* **35**(2), 132–141 (2000). 11021338 10.1007/BF02688772

[58] Butkevich, I. P., Mikhailenko, V. A. & Leont’eva, M. N. Sequelae of prenatal serotonin depletion and stress on pain sensitivity in rats. *Neurosci. Behav. Physiol.* **35**(9), 925–930 (2005). 16270174 10.1007/s11055-005-0147-5

[59] Wiesenfeld-Hallin, Z. Sex differences in pain perception. *Gend. Med.* **2**(3), 137–145 (2005). 16290886 10.1016/S1550-8579(05)80042-7

[60] De Angelis, F. *et al.* Sex differences in neuropathy: The paradigmatic case of metformin. *Int. J. Mol. Sci.* **23**, 23 (2022). 10.3390/ijms232314503 PMC9738696 36498830

