# Author Correction: *Ganoderma tsuage* promotes pain sensitivity in aging mice

**DOI:** 10.1038/s41598-024-72064-0

**Published:** 2024-09-10

**Authors:** Kai-Ning Yang, Chia-Ying Lin, Wei‑Nong Li, Chao‑Ming Tang, Jyotirmayee Pradhan, Ming‑Wei Chao, Chia‑Yi Tseng

**Affiliations:** 1 Taoyuan General Hospital, Ministry of Health and Welfare, Taoyuan, Taiwan; 2 Department of Biomedical Engineering, Chung Yuan Christian University, Taoyuan, Taiwan; 3 Department of Bioscience Technology, Chung Yuan Christian University, Taoyuan, Taiwan

Correction to: *Scientific Reports* 10.1038/s41598-024-61499-0, published online 21 May 2024 

The original version of this Article contained an error in Figure 5, where there was an error in the legend of the y axis:

“Time spent in pain related behaviors (sec)” now reads “No. of paw licking”

Also there was an error in the caption of Figure 5:

“Formalin test to assess pain reaction in the right paw of mice upon D-gal-induced aging. In phase I (0–10min), the number of mice licking the paw increased in the D-Gal aging group compared to the control. In phase II (10–30min), the number of paw licking in the GT group significantly increased compared with the control. The number of paw-licking events in the aging group significantly decreased, which could be reversed by GT. N = 6 except control (N = 5).

now reads:

“Formalin test to assess pain reaction in the right paw of mice upon D-gal-induced aging. In phase I (0–10min), the number of mice licking the paw increased in the D-Gal aging group compared to the control. In phase II (10–30min), the number of paw licking in the GT group significantly increased compared with the control. The number of paw-licking events in the aging group significantly decreased, which could be reversed by GT. *p < 0.05, **p < 0.01 compared to the control of phase I using Student’s t-test and #p < 0.05, ##p < 0.01 compared to the control of phase II by Student’s t-test. N = 6 except control (N = 5)”

The original Article has been corrected.

